# Demographic variation in nutrition knowledge in England 

**DOI:** 10.1093/her/15.2.163

**Published:** 2000-04

**Authors:** K. Parmenter, J. Waller, J. Wardle

**Affiliations:** ICRF Health Behaviour Unit, Department of Epidemiology & Public Health, University College London, 2–16 Torrington Place, London WC1E 6BT, UK

## Abstract

This paper describes a nutrition knowledge survey carried out on a cross-section of the adult population of England (*n* = 1040), looking at knowledge relating to current dietary recommendations, sources of nutrients, healthy food choices and diet–disease links. Serious gaps in knowledge about even the basic recommendations were discovered, and there was much confusion over the relationship between diet and disease. Significant differences in knowledge between socio-demographic groups were found, with men having poorer knowledge than women, and knowledge declining with lower educational level and socio-economic status. Possible reasons for these differences and implications for public education campaigns and socio-economic inequalities in health are discussed.

## Introduction

In the richer countries of the world at the end of the 20th century, consumers may be literally `spoiled for choice' in the food domain. They must select their foods from hundreds or even thousands of products, many of which are designed and marketed to maximize their appeal to the consumer. To select a healthy diet, they must be able to ignore the advertisers' blandishments and the immediate appeal to the palate, and draw on a complex technical and scientific knowledge base concerning nutrients, foods and health. At a minimum, they need to know the prevailing nutritional recommendations, be able to apply those to the food products which they are considering and combine recommendations to make the best food choices.

Nutritional recommendations are published regularly in most countries (Cannon, 1992), together with public information aimed at helping people to understand the links which have been established between diet and disease (WHO, 1990). However, the results of surveys which examine the public understanding of nutritional advice have not been encouraging. In the UK, the Health Education Monitoring Survey (HEMS) was carried out to establish how successfully the recommendations put forward in the Department of Health's *The Health of the Nation* White Paper (Department of Health, 1992) were being disseminated (Hansbro *et al.*, 1997). When asked to describe what they understood by a healthy diet, only 16% of respondents mentioned as many as three out of four of the core recommendations (to eat more fruit, vegetables and salad; to cut down on fat; to eat more fibre; to eat more starchy carbohydrate). Although these were the most common answers, they were given by only 67, 45, 27 and 20% of respondents, respectively. Over two-thirds of respondents (68%) endorsed the statement that `experts never agree about what foods are good for you', demonstrating that clear messages about healthy eating are not being conveyed to the general public. The ignorance about nutritional advice was mirrored by little will to make healthy changes. Although more than 80% of people believed that their diets could be healthier [a larger number than has been found elsewhere, e.g. (de Graaf *et al.*, 1997)], two-thirds either did not want to change or wanted to but did not think that they would.

Studies in the UK looking at other aspects of nutrition knowledge have had equally concerning findings. Buttriss (Buttriss, 1997) found that people were poor at identifying foods containing starch and even worse at knowing which foods contained fibre, with 35% of the sample failing to correctly categorize as many as half of the foods presented as high or low in fibre. Tate and Cade (Tate and Cade, 1990) reported positive findings regarding knowledge of dietary fat and coronary heart disease. However, there were areas of knowledge which were extremely poor (e.g. 84% of people believed that sunflower margarine contains less fat than butter). Although the majority of items were answered correctly by at least 70% of people, this still means that over a quarter of respondents were incorrect.

Research in other countries has reported similar results. Analysing surveys conducted in the USA in 1983, 1986 and 1988, Levy *et al.* (Levy *et al.*, 1993) found that although nutrition knowledge relating to fat and cholesterol was improving, it was still unacceptably low, with no more than 60% of respondents correctly answering any individual item. Patterson *et al.* (Patterson *et al.*, 1995) found that 23% of Americans were not aware of *any* of the National Cancer Institute's dietary recommendations, and 36% believed links between diet and cancer to be weak or non-existent. Cotugna *et al.* (Cotugna *et al.*, 1992), in a larger US survey, found that 52% of those asked about diet–disease links did not mention cancer unprompted, and 27% still did not acknowledge any link between diet and cancer when asked specifically about it. True levels of knowledge may even be lower than those reported, as survey response rates tend to be around 70% and it is probable that non-responders would have lower knowledge than responders.

People with poorer nutrition knowledge may fall into particular socio-demographic groups. In the HEMS (Hansbro *et al.*, 1997) mentioned above, women had better knowledge than men, and knowledge tended to be better at higher levels of education and socio-economic status (SES). These findings are in keeping with other research. In the US, Levy *et al.* (Levy *et al.*, 1993) found that levels of knowledge about fat and cholesterol were highest for more educated people, people of middle years and White people. In a study in Australia, Crawford and Baghurst (Crawford and Baghurst, 1990) found that knowledge about the links between disease and fat, sodium and sugar was generally better for women, people of higher SES and older people, and Tate and Cade (Tate and Cade, 1990) reported similar results from a UK sample. Buttriss (Buttriss, 1997), also in the UK, found significant gender and class differences in ability to identify foods containing starch and fibre, with women performing better than men, and middle and upper-middle class respondents better than working class respondents.

To summarize, then, studies have tended to find women more knowledgeable than men (Crawford and Baghurst, 1990; Tate and Cade, 1990; Buttriss, 1997; Hansbro *et al.*, 1997), knowledge to increase with higher SES (Tate and Cade, 1990; Buttriss, 1997; Hansbro *et al.*, 1997) and education (Cremer and Kessler, 1992; Levy *et al.*, 1993; Hansbro *et al.*, 1997), and generally to be better in middle aged than older or young groups (Anderson *et al.*, 1988; Tate and Cade, 1990; Levy *et al.*, 1993; Hansbro *et al.*, 1997), although the patterns found across age groups have not always been the same.

Existing studies of nutrition knowledge in the UK have tended to focus on one particular aspect of nutrition, e.g. fat, fibre, etc., rather than taking a broad perspective. There has also been a tendency to use *ad hoc* measures with little attention to issues of reliability and validity. This has limited our understanding of the state of nutrition knowledge in the UK.

Most of the studies looking at demographic differences in nutrition knowledge and dietary behaviour have used occupation as a measure of SES and have not taken into account differences in education. Given that SES is a complex construct including economic status (income), social status (education) and work status (occupation) (Adler *et al.*, 1994), it is important to try to establish the contribution of different socio-economic variables to the variation in knowledge.

The aim of the present study was to examine nutrition knowledge and demographic variations in knowledge, in a wide cross-section of the adult population of England. Knowledge was assessed using the recently developed and validated Nutrition Knowledge Questionnaire (Parmenter and Wardle, 1999). This instrument covers knowledge relating to current dietary recommendations, sources of different nutrients, everyday food choices and diet–disease links. Demographic characteristics were assessed in the same instrument with simple questions.

## Subjects and methods

### Subjects

As the majority of the people in England are registered with a general practitioner (GP), GP practices were recruited to participate in the study in the hope of reaching a wide cross-section of the population. Three practices were used, one each in Essex, Lancashire and Oxfordshire, thereby giving cluster samples from very different areas of the country. Five hundred patients aged 18–75 were selected at random from each of the patient lists (750 men and 750 women in total) and contacted by post with a request to take part in the survey.

### Materials

Nutrition knowledge was assessed using the Nutrition Knowledge Questionnaire, the development of which is described elsewhere (Parmenter and Wardle, 1999). The questionnaire has four sections covering (1) experts' recommendations regarding increasing and decreasing intake of different food groups, (2) nutrient knowledge, (3) food choice (which asks people to choose between different options, e.g. to pick the snack which is low in fat and high in fibre), and (4) the relationships between diet and disease. This last section looks at beliefs about which foods can cause particular diseases, as well as knowledge of any diseases associated with eating too much or too little of various types of food.

Demographic questions covered age, gender, ethnic origin, work status, occupation and partner's occupation, level of education, marital status, number of children, and children under 18 living at home. Questions were also asked about specialist nutritional training and specific dietary requirements.

### Procedure

Each participant was written to personally in a letter from their GP asking them to help with a study which was described as looking at `people's understanding of nutrition advice'. The questionnaire was enclosed, together with a postage paid envelope for returning it. A second letter and another questionnaire was sent to anyone who had not returned the questionnaire after 2 weeks, in an attempt to maximize the response rate (Dillman, 1991).

## Results

### Sample

A response rate of 73.6% (*n* = 1040) was achieved, comprising 43.8% men and 56.2% women.

The mean age of respondents was 51.5 years and the majority of respondents were White. Socio-economic status followed a normal distribution, although a large number of people could not be classified on the basis of the occupational information which they supplied. The level of education followed a straight line, with numbers decreasing as educational level increased. The demographic characteristics of the sample are shown in Table I.

Comparison with the demographic characteristics of the UK population from the 1991 Census (Office of Population Censuses and Surveys, 1991) showed that the sample was biased in favour of women, people of high SES and educational qualifications, White people, and older age-groups. These differences, typical of postal surveys of this sort, obviously limit the degree to which results can be generalized to the English population as a whole and the implications of this will be discussed in more detail later. However, the sample was sufficiently large to allow trends in knowledge across the demographic characteristics to be clearly identified.

### Nutrition knowledge

**Fig. 1.**
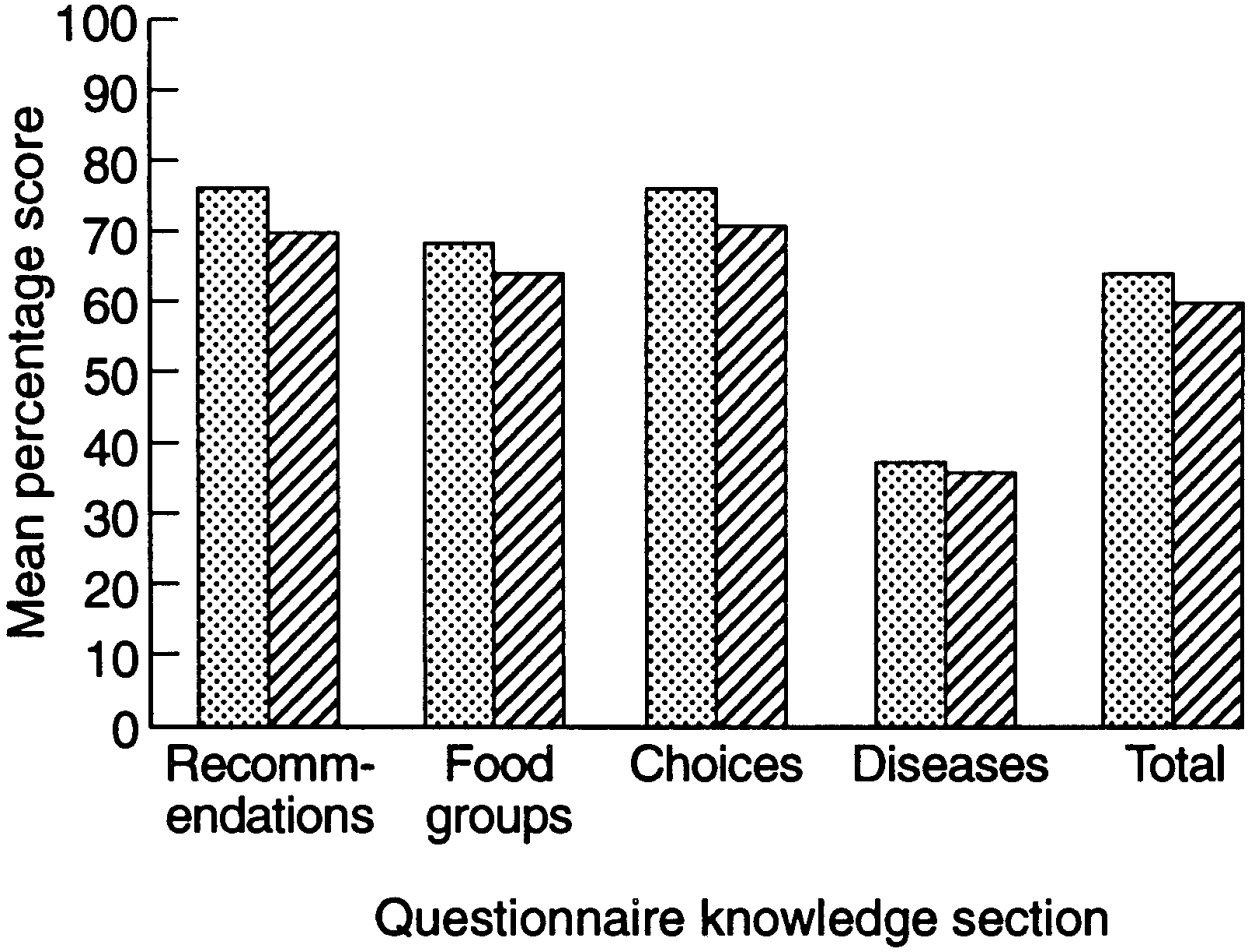
Percentage correct on each section for men and women; ▪ indicates women, ▪ indicates men.

Figure 1 shows the mean percentages of correct responses for all sections and the whole questionnaire.

#### Section 1—dietary recommendations

Out of a maximum of 11 points for the first section, the mean score was 8.1 (SD 1.80). More than 90% of respondents were aware of the recommendations to decrease fat, sugar and salt intake, and increase fibre, fruit and vegetables, indicating that these basic messages are being successfully conveyed. However, almost a quarter did not know the recommendation to reduce saturated fat. Half of respondents (51%) were not aware of advice to cut down on meat and almost 90% were unaware of the recommendations to eat more starchy carbohydrates. Seventy percent did not know that the recommended daily intake of fruit and vegetables was as many as five or six servings, with just over 50% believing one to three portions to be adequate.

#### Section 2—food groups

Of a possible 69 points for the section on food groups, the mean score was 45.6 (SD 11.63). When asked to categorize various foods as either high or low in sugar, fat, starch, salt, protein, fibre or saturates, mistakes were generally made by just under a third of respondents. There were, however, a few items which stood out as being particularly poorly answered. Eighty-five percent of people failed to realize that low-fat spread is actually high in fat, with the majority of respondents believing it to be a low-fat food. Only just over half of respondents knew that nuts are low in starch and fewer than half realized that cheese is high in salt.

The section on fibre was generally well answered. This represents a substantial improvement on the findings of Buttriss (Buttriss, 1997), mentioned above, and Cremer and Kessler (Cremer and Kessler, 1992) who found people's knowledge about which foods contained fibre to be significantly lower than equivalent knowledge about fat.

People were generally better at identifying foods which are high in saturated fat than those which are low in it. There was much confusion about whether foods could be high in fat but contain no cholesterol (agree = 27%, disagree = 29%, not sure = 44%). Over 70% of respondents either incorrectly believed that margarine contained less fat than butter or were unsure. Knowledge about monounsaturated fat was also poor, with fewer than a quarter of people knowing that olive oil contains mostly this type of fat. Finally, people were confused about which food types contain most energy. Almost equal numbers believed it was fatty and sugary foods (33 and 35%, respectively), with 22% being unsure.

#### Section 3—everyday food choices

Out of a maximum of 10 points on this section, the mean score was 7.4 (SD 1.83). Responses to the different items varied widely, seeming to depend largely on the distracter items. The most mistakes were made on the question which asked people to pick a low-fat, high-fibre snack. Only 36% chose the correct answer (raisins), with almost half (47%) endorsing the muesli bar distracter option. As many as a third of respondents were unable to select the best choice for a low-fat cheese and approximately 30% of people did not know that thick cut chips are `healthier' than thin or crinkle cut ones.

#### Section 4—diet–disease relationships

In this section participants were asked first whether they knew of any links between eating more or less of particular foods and major health problems. The mean score was 7.35 (SD 3.41) out of a possible 20 points. The highest proportion of people were aware of a relationship between high fat intake and disease, but almost 15% of people still did not know about this link. Of the people who were aware of the fat–disease link, over 90% also knew about the link between saturated fat and heart disease.

As regards fruit and vegetables, well over a third of respondents (41%) were unaware of a link between low intake and health problems. Only 42% correctly thought that eating more fruit and vegetables can help reduce the risk of cancer, and 47% knew that it could also reduce the chances of heart disease.

Approximately two-thirds of respondents (62.1%) knew of health risks associated with a low fibre intake, with the majority of these people being aware of the specific risk of cancer.

Over 60% of people were also aware of links between sugar and salt intake and disease; 84% knew of the link between a high salt diet and heart disease.

When asked to specify diseases which were linked with different food types, respondents' answers varied enormously. For fruit and vegetables, the most commonly mentioned disorders were scurvy and bowel problems, with answers ranging from varicose veins to Beriberi. Bowel problems were also associated with insufficient fibre by many people, with the most commonly mentioned disorders being bowel problems/cancer and constipation. Most people thought that sugar could cause diabetes and obesity, but only about a quarter mentioned tooth decay. High blood pressure and heart disease were associated with excessive salt intake by 57 and 43% of the respondents who answered the item. Finally, 81% of people mentioned heart problems as being associated with high fat intake, with overweight/obesity being the second most popular response.

The poorest scores in this section concerned antioxidant vitamins with only 22% of respondents having heard of them. When these people were asked to say whether a particular vitamin was an antioxidant, less than half of them gave the correct answer on any item.

### Demographic differences in knowledge

For the purposes of analysis, age was divided into five groups and educational level into four. Marital status was categorized as single, married or living as married, and separated, divorced or widowed. Ethnic origin was not used in the analyses as the vast majority of respondents were White. Fewer than 6% of people had nutrition-related qualifications and fewer than 10% were on special diets, so these factors were also omitted from analyses.

#### Univariate analyses

As predicted, women scored slightly, but significantly higher than men on the knowledge questionnaire as a whole (*t*[d.f. = 1037] = 4.86, *P* < 0.001) and on each of the sections individually. The mean percentage of correct responses for men and women for each section are shown in Figure 1.

**Fig. 2.**
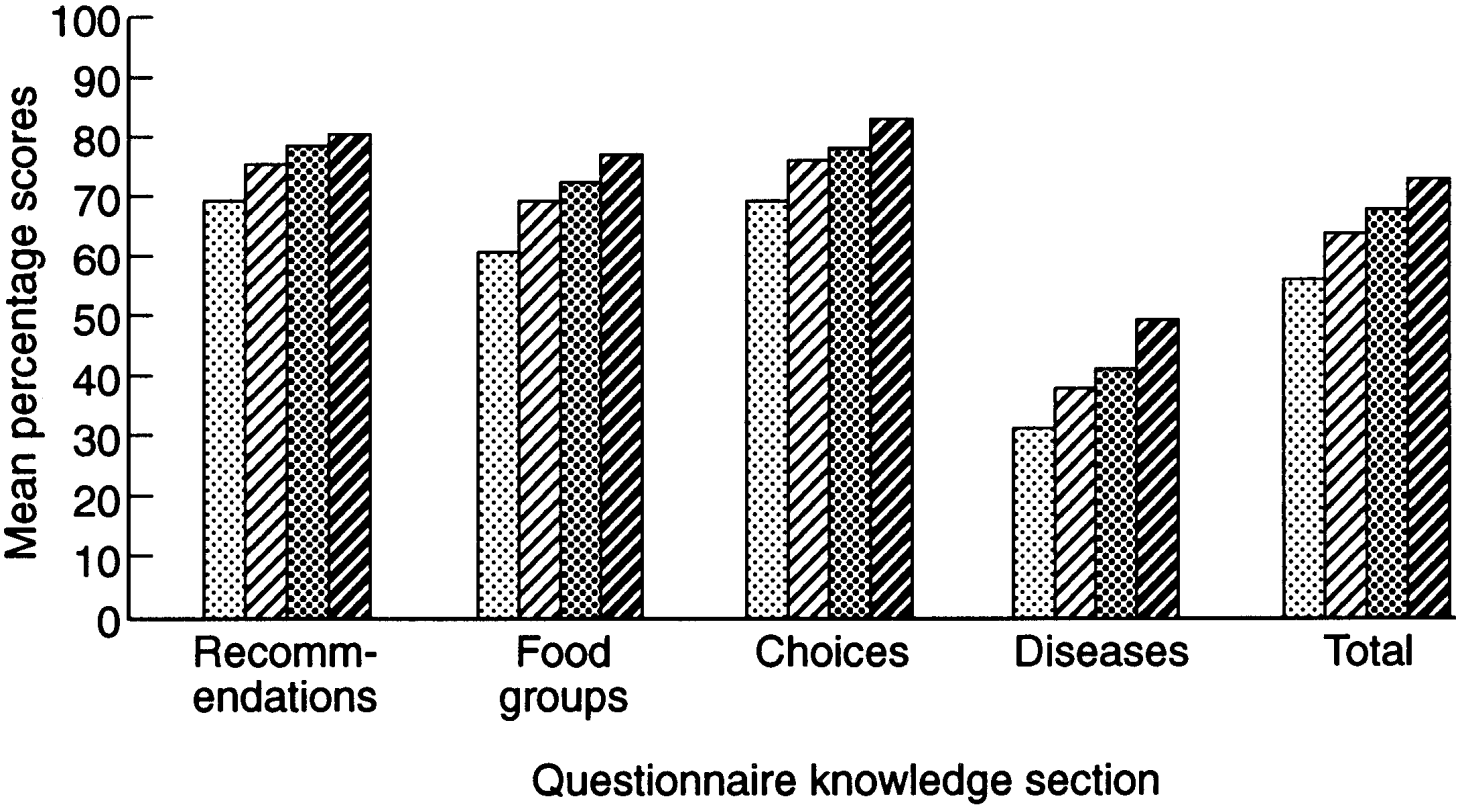
Knowledge differences across educational levels. From left to right, bars represent `No qualifications', `O level', `A level' and `Degree'.

Figure 2 shows that there was a linear relationship between knowledge and education level, with scores being lowest for people with no formal qualifications while those with degrees scored highest (*F*[4,1009] = 49.1, *P* < 0.001 for total score).

**Fig. 3.**
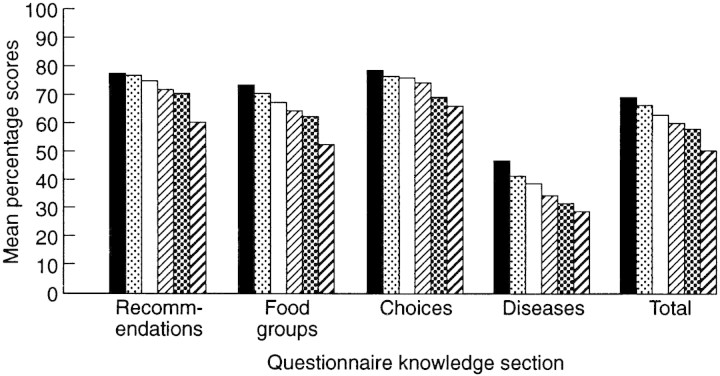
Knowledge differences across SES groups. From left to right, bars represent classes `I', `II', `III non-manual', `III manual', `IV' and `V'.

A similar pattern was found for social class defined on the basis of occupation (see Figure 3) with total scores being lowest for those in class V, rising progressively to class I (*F*[5,810] = 16.2, *P* < 0.001). It should be noted that there was a large number of missing values for SES (where occupational information was insufficient for classification), so only about 77% of cases could be used for analyses including this variable.

**Fig. 4.**
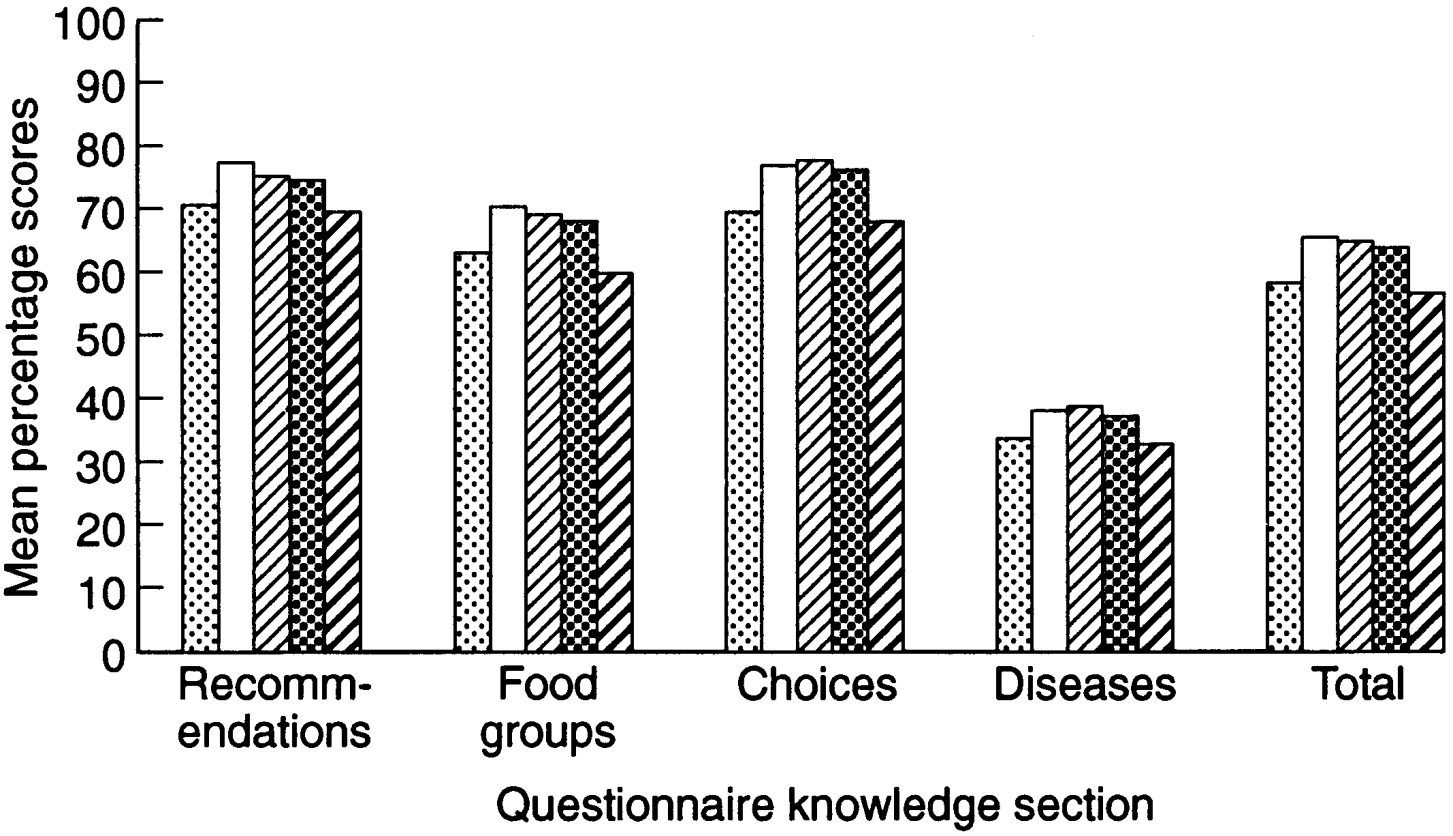
Knowledge differences across age groups. From left to right, bars represent the following age groups: `18–34', `35–44', `45–54', `55–64' and `65 and over'.

As illustrated by Figure 4, the youngest age group scored lower than people in middle years, with those aged over 65 obtaining the lowest scores. One way analysis of variance showed these differences to be significant (*F*[4,1038] = 16.2, *P* < 0.001 for total score).

Respondents who were married or living as married achieved slightly higher scores (mean percentage correct = 63.1%) than those who were either single (58.8%) or separated, divorced or widowed (58.9%). One-way analysis of variance showed the differences between these groups to be significant (*F*[2, 1036] = 7.5, *P* < 0.001).

Respondents with children living at home scored significantly higher (64.2%) than those without (61.3%) (*t*[d.f. = 1038] = 2.85, *P* < 0.01).

#### Multivariate analyses

In order to establish whether education, gender, SES, marital status and the presence of children at home were all having separate effects on knowledge score, these variables were entered into a multiple regression model. Marital status was recoded to married/cohabiting versus not married. The results showed that gender, level of education and occupational social class all had significant independent effects at the 0.01 level, and the effect of marital status was significant at the 0.05 level (see Table II). Together these variables accounted for 22% of the variance in knowledge score.

The curvilinear relationship between age and knowledge score meant that it was not appropriate to enter age into the linear regression model. The distribution of respondents across age groups was very similar for men and women, indicating that its effect was not confounded with that of gender. There were, however, different patterns for educational level and SES across age, with level of education tending to decrease with increasing age and SES being higher for people of middle years than for the oldest or youngest groups. In order to try to establish whether age was having an independent effect on knowledge score, the sample was stratified by education and SES. The largest educational subgroup was those people with no educational qualifications (*n* = 431). If variation of score with age in this subgroup mirrored that of the whole sample, it was likely that age was having an independent effect. This was indeed the case, with significant differences between age groups for total score (*F*[4,431] = 7.48, *P* < .001) and scores on the individual sections (all *P* < 0.05). The pattern was the same for the group with O levels (*n* = 281) (*F*[4,280] = 8.49, *P* < 0.001), but the effect disappeared for the A level and degree groups, indicating that age might only have an effect on knowledge among people with lower levels of education. It should, however, be noted that the numbers of respondents in these two groups were smaller (*n* = 166 and 133, respectively), reducing the power of the ANOVA.

The same analysis was carried out stratifying by SES and the age pattern persisted in each SES group. The effect was significant for SES group II, which was the largest (*F*[4, 235] = 3.74, *P* = 0.006).

## Discussion

The results of this survey give a clear and detailed picture of a broad range of nutrition knowledge in a large sample of the English general public. The response rate (over 70%) was excellent for a mailed survey of this kind. Nevertheless, we have to be cautious in extrapolating beyond this respondent sample. As mentioned earlier, our sample, though large, was not wholly representative of the general population. It was biased in favour of women, older people, high SES and education, and White people, probably reflecting differential response rates by different demographic groups. Previous research (e.g. Anderson *et al.*, 1988; Tate and Cade, 1990; Cremer and Kessler, 1992; Buttriss, 1997; Hansbro *et al.*, 1997) has shown that these groups tend to have better nutrition knowledge than the general population, so it is probable that our results over-estimate the level of knowledge in England as a whole.

The results are partly encouraging, with many respondents being aware of most of the major guidelines on healthy eating. The results look better than those obtained in the HEMS (1997), which could be attributable to the format of the questionnaire. People seem to be poor at spontaneously generating guidelines for healthy eating, as they were required to do in the HEMS (1997), but when asked about specific recommendations, they generally appear to know whether they should be eating more or less of particular types of food. The main exception to this was carbohydrate, indicating that more effort is needed to raise awareness of the importance of increasing intake of starchy foods. The question remains whether spontaneous recall or recognition would be most influential in everyday life.

On the second section of the questionnaire, the greatest confusion in categorizing foods as high or low in different nutrients concerned low-fat spreads. Many people made the mistake of classifying these as low in fat. This could be because people genuinely believe these spreads to be low in fat, but it also seems possible that when quickly looking down a list of foods, and ticking boxes, it might be almost automatic when faced with `low-fat spreads' to tick the box marked `low fat'. Confusion may arise because people know that these foods are *lower* in fat than, for example, butter. This could be clarified by asking people to classify the spreads into a particular food group rather than just saying whether they were high or low in fat.

Generally people performed fairly well on the food choice section indicating that they can translate their knowledge into actual choices. The most common mistake was choosing a muesli bar as a low-fat, high-fibre snack. This might be attributable to marketing, presenting an image of muesli bars as a `healthy' alternative to more fattening snacks. The pervasiveness of the error could indicate that advertising is used by many people as a source of nutrient information and that people do not actually read the nutritional information labels on foods to find out their fat or fibre content. This would tie in with the HEMS (1997) research which found that large numbers of people never look at the ingredients of foods when shopping. More research is needed to clarify the effects of marketing on beliefs about nutrition.

Knowledge about diet and disease was poor. Most people were inclined to believe in links between all sorts of foods and a huge variety of diseases. The only really well-known relationships seemed to be between high fat and salt intake and cardiovascular disease, although approximately one in five people were still unaware of these links. Other than that, it seems that people are very confused about the effects of different foods on their health. This is in keeping with other research. For example, Anderson *et al.* (Anderson *et al.*, 1998) found people in England and Scotland had poor knowledge about links between fruit and vegetables and cancer. Similarly, Krebs-Smith *et al.* (Krebs-Smith *et al.*, 1995) found that only 40% of adults in the US agreed with the statement that eating fruit and vegetables prevents cancer. The worst-answered items in this section were those relating to antioxidant vitamins. Perhaps not surprisingly, given the recency of scientific findings relating to them, only about one in five people had heard of them and very few knew which vitamins were classified as antioxidant.

This study had the advantage of a substantial sample and so although it was not entirely representative of the general population, groups were large enough so that multivariate analyses could be used to establish whether the demographic variables exerted independent effects. Women demonstrated superior knowledge of all areas of nutrition, as has been found in most studies looking at nutrition knowledge. The gender difference does not seem to have diminished significantly over the last 10 years. With the decline in the number of traditional family units where the husband earns and the wife is responsible for shopping and cooking, and the rise in the number of people living alone (Bridgwood and Savage, 1993), it is increasingly important for men to know how to eat healthily. So far, it appears that even though more men are cooking for themselves and fewer are relying on women to make decisions about their diets, this is not accompanied by an increase in nutrition knowledge, highlighting the need to target men in education campaigns. Articles relating to diet are still very much the domain of women's magazines, something which must change if men are to learn more about eating healthily. More research would be needed to identify effective media for conveying messages about healthy eating to men.

The second major demographic trend involved level of education, with more educated people demonstrating significantly better nutrition knowledge. This may be because education incorporates the very information that is included in this survey, but as many nutritional recommendations are relatively recent (e.g. eating five portions of fruit and vegetables every day), yet are still better known by more educated respondents, this cannot be the whole explanation. People who are better educated may also be better able to make use of written material like newspaper articles and leaflets, to gain information and implement it in their lifestyles. It also seems likely that more educated people would be better able to understand the sometimes complex information about diet–disease links. SES was also shown to have an effect independent of educational qualifications, suggesting that aspects of the social and cultural milieu might modify exposure to nutritional advice.

Much nutrition knowledge (e.g. the need to reduce fat intake) is now assumed to be very widely held. This has the effect that many newspaper articles, television programmes and other forms of popular journalism assume a certain level of knowledge which, though common to most people, appears not to be reached by everyone. This might be a particular problem for less educated or low SES people, making some sources of nutrition information completely inaccessible due to lack of background knowledge and inability to deal with the new information. If this is the case, ways need to be found of targeting basic information at certain groups of people to bring their level of knowledge up to that of the rest of the population. Once again, more research is needed to find out which methods are effective for communicating to different groups.

The variation in knowledge with age was less clear-cut. Different studies have found different patterns, but it is often true that the middle-aged group perform best, as was found in the present study. The poor scores of the oldest group probably reflect the fact that the current dietary recommendations are relatively recent and older people probably have more established views on food. In the first half of the century when these people were growing up, the government was encouraging people to eat high fat, high sugar diets (Cannon, 1992), so it seems understandable that older people are less receptive to new guidelines which are directly contrary to this. Low scores in the youngest group might be indicative of a lack of interest in health care issues. One would expect that as people reach middle age they become increasingly aware of diseases related to diet as they or their peers are affected by them and information about nutrition becomes more salient. This increased knowledge might also be associated with having children and seeking out dietary information to ensure that the children are eating healthily. However, to gain a true understanding of the relationship between age and knowledge, it would be necessary to conduct longitudinal research to differentiate between cohort effects and changes in knowledge related to life stages.

These demographic patterns have some parallels in dietary behaviour. The Dietary and Nutritional Survey of British Adults (Gregory *et al.*, 1990) found that women were more likely to eat healthy foods like wholemeal bread, fruit, vegetables and reduced-fat milk, although they also ate more confectionery. Men, on the other hand, reported eating more sausages, meat pies and chips. This indicates that poorer nutrition knowledge in men could be being translated into less healthy eating habits.

In further analysis of the survey data (MAFF, 1994), men and women of higher SES reported eating more fruit, fruit juice, vegetables, salad, polyunsaturated margarine, oily fish and shell fish, although they also ate more buns, cakes and chocolate, suggesting that knowledge differences between SES groups are also mirrored by dietary variations.

Other studies have looked more generally at lifestyle patterns, including diet, and have found similar demographic variations. Whichelow and Prevost (1996) grouped dietary behaviour into four components, one of which correlated with high intakes of fruit and vegetables, high-fibre foods and low-fat spreads and milk. This component (the one which most closely meets current dietary recommendations) was favoured by those of middle years rather than the very old or very young. It was also linearly associated with SES, being most popular with the professional group. When the sample was divided into manual and non-manual groups, women from each group scored higher on this component than men of the same SES. This patterning has striking similarities with the variations in knowledge found in the present study. In a follow-up study of the same sample 7 years later (Prevost and Whichelow, 1996), there was a significant increase in scores for the healthy diet component. However, non-manual responders showed significantly greater health-related improvements than those with manual occupations, indicating that differences in diet across socio-economic groups are persisting and even widening.

Knowledge is not, of course, the only factor underlying dietary behaviour. Other important influences include the quality and freshness of the food, taste, price and family preferences (Lennernäs *et al.*, 1997). The relative importance of these factors seems to vary with demographic characteristics. In their pan-European survey, Lennernäs *et al.* (Lennernäs *et al.*, 1997) found that men tend to rate taste as more important than eating healthily. Eating healthily is regarded as a priority by people with tertiary education, whereas price is more salient for those with only primary education. Price, taste and habit were identified as important barriers to change. However, these influences may to some degree be underpinned by knowledge. Dowler and Calvert (1995) found that among lone-parents on very low incomes, those who looked for fresh food and aimed to give their children a healthy diet consistently achieved a healthier diet (albeit within their financial constraints) than those who did not. This indicates that nutrition knowledge could be important even when other barriers and constraints are present.

The implications of this research are two-fold. Firstly, the indications are that an unacceptably large number of people are still unaware of the main dietary recommendations, as well as having poor knowledge about the sources of nutrients and diet–disease links, and being unable to make healthy food choices. Given the demographic biases in our sample, the real picture in the UK is likely to be worse than is suggested by the present results. This has implications for public education campaigns. Awareness of the need to eat five portions of fruit and vegetables every day, and the need to increase carbohydrate must be raised. Clarification about the fat content of margarine and low-fat spreads is needed, as is clear information about the links between diet and disease, over which there still seems to be a great deal of confusion. It must also be remembered that a significant proportion of people remain unaware of the main guidelines, including the need to reduce fat, sugar and salt intake, and to increase fruit and fibre intake.

Secondly, the demographic differences in knowledge must be addressed. More research is needed to gain a clearer understanding of nutrition knowledge in those groups which were under-represented in our sample, especially ethnic minority groups and those with low educational qualifications and SES. Although it might be argued that knowledge plays a limited part in food choice, the demographic patterning of food choice in the Dietary and Nutritional Survey of British Adults (Gregory *et al.*, 1990) and the HEMS (Hansbro *et al.*, 1997) is remarkably similar to the demographic differences in knowledge observed in the present study, and it is at least probable that deficiencies in knowledge contribute to deficiencies in diet. Furthermore, dietary inequalities may even be increasing, which may partly rest on the growing availability of nutritional advice to which more privileged groups seem to have more access. By targeting education at those who particularly need it we can help to reduce the divides which lead to the perpetuation of inequalities in health. Knowledge was identified by Link and Phelan (Link and Phelan, 1996) as one of the `fundamental social causes' of differences in health, so by empowering people with the knowledge to make appropriate dietary decisions, we can take a step towards ending this pattern of social inequality.

## 

**Table I.** *Demographic characteristics of the sample (*n *= 1040)*

	*n*	%
Gender		
male	455	43.8
female	584	56.2
Age		
18–34	143	13.8
35–44	208	20.0
45–54	237	22.8
55–64	212	20.4
65 and over	239	23.0
Ethnicity		
White	1033	99.4
other	6	0.6
Marital status		
married/living as married	809	77.9
single	102	9.8
widowed/divorced/separated	128	12.3
Educational level		
no qualifications	431	42.5
O level/equivalent	281	27.7
A level/equivalent	169	16.7
degree/higher degree	133	13.1
Employment status		
employed full-time	393	38.0
employed part-time	177	17.1
full-time homemaker	110	10.6
retired	283	27.4
other	70	6.8
SES		
I	77	7.4
II	236	22.7
III non-manual	172	16.5
III manual	162	15.6
IV	135	13.0
V	29	2.8
not ascertained	229	22.0
Children living at home?		
yes	309	29.7
no	730	70.3

**Table II.** *Multiple regression of overall nutrition knowledge score on gender, education, SES, marital status and presence of children at home*

Variable	β	*P*	
Gender	–0.23	<0.001	multiple *R* = 0.466
Education	–0.34	<0.001	adjusted *R*^2^ = 0.212
SES	–0.12	0.002	*F*[5,799] = 44.33, *P* < 0.001
Marital status	0.08	0.015	
Children at home	–0.04	0.225
